# A family of silicon transporter structural genes in a pennate diatom *Synedra ulna* subsp. *danica* (Kütz.) Skabitsch

**DOI:** 10.1371/journal.pone.0203161

**Published:** 2018-08-29

**Authors:** Artyom M. Marchenkov, Darya P. Petrova, Alexey A. Morozov, Yulia R. Zakharova, Michael A. Grachev, Alexander A. Bondar

**Affiliations:** 1 Department of Cell Ultrastructure, Limnological Institute, Siberian Branch of the Russian Academy of Sciences, Irkutsk, Russia; 2 Genomics Core Facility, Institute of Chemical Biology and Fundamental Medicine, Siberian Branch of the Russian Academy of Sciences, Novosibirsk, Russia; UMR-S1134, INSERM, Université Paris Diderot, INTS, FRANCE

## Abstract

Silicon transporters (SIT) are the proteins, which capture silicic acid in the aquatic environment and direct it across the plasmalemma to the cytoplasm of diatoms. Diatoms utilize silicic acid to build species-specific ornamented exoskeletons and make a significant contribution to the global silica cycle, estimated at 240 ±40 Tmol a year. Recently *SaSIT* genes of the freshwater araphid pennate diatom *Synedra acus* subsp. *radians* are found to be present in the genome as a cluster of two structural genes (*SaSIT-TD* and *SaSIT-TRI*) encoding several concatenated copies of a SIT protein each. These structural genes could potentially be transformed into “mature” SIT proteins by means of posttranslational proteolytic cleavage. In the present study, we discovered three similar structural *SuSIT* genes in the genome of a closely related freshwater diatom *Synedra ulna* subsp. *danica*. Structural gene *SuSIT1* is identical to structural gene *SuSIT2*, and the two are connected by a non-coding nucleotide DNA sequence. All the putative “mature” SITs contain conserved amino acid motifs, which are believed to be important in silicon transport. The data obtained suggest that the predicted “mature” SIT proteins may be the minimal units necessary for the transport of silicon is *S*. *ulna* subsp. *danica*. The comparative analysis of all available multi-SITs has allowed us to detect two conservative motifs YQXDXVYL and DXDID, located between the “mature” proteins. Aspartic acid-rich DXDID motif can, in our opinion, serve as a proteolysis site during the multi-SIT cleavage. The narrow distribution of the distances between CMLD and DXDID motifs can serve as additional evidence to the conservation of their function.

## Introduction

Silicon (after oxygen) is the second most abundant element on the planet, amounting to 28% of Earth's crust by mass [1]. In water environments it is available in the form of dissolved silicic acid (H_2_SiO_4_) [2], mostly delivered to water bodies by river inflow, direct atmospheric deposition and/or biogenic silica dissolution. Essentially, via diatom biomineralisation and on geological timescales via continental weathering [3]. The silica cycle was shown to be tightly bound to that of carbon [4, 5]. Silicic acid is an important biogenic element that a variety of organisms with use to build different siliceous structures or to strengthen their cell walls, including diatoms, chrysophytes, metazoans, silicoflagellates, radiolarians, choanoflagellates, embryophytes and other organisms [6–8].

In particular, diatoms (Bacillariophyta) are one of the most numerous and ecologically important groups of phytoplankton. Via the use of silicic acid, they form ornate species-specific exoskeletons [9] and globally assimilate 240±40 Tmol of silicic acid a year [10], and providing up to 20% of the Earth’s primary organic carbon production [11].

Critical for biosilicification is the process of capture and transport of silicic acid from the aquatic environment through the plasmalemma. Silicic acid is transported into the cell by SITs (silicon transporters) against a concentration gradient. The molecular mechanism of such transport remains unclear.

The first *SIT* gene encoding a silicon transporter protein was found in a marine pennate raphid diatom *Cylindrotheca fusiformis* [12]. The genome of this diatom appears to contain five distinct homologous *SIT* genes. SITs are a family of membrane proteins. The *C*. *fusiformis* SIT contains 10 transmembrane domains [13]. It was demonstrated that SIT proteins contain a unique, highly conserved extra-membrane motif with the consensus sequence of CMLD [14, 15]. The next diatom in which *SIT* structural gene (AF492011) was found was the freshwater pennate araphid diatom *Synedra acus* subsp. *radians* from Lake Baikal [14]. Since then, *SIT* genes have been found in many species of diatoms [16–20], as well as in some other silicifying organisms: chrysophytes [21], choanoflagellates [22] and haptophytes [23]. Thamatrakoln et al. revealed four conserved GXQ motifs in the predicted SIT amino acid sequences [24]. They supposed that these motifs could be involved in silicic acid transport. Diatom *SIT*s were shown to form five clades, A to E, in the phylogenetic tree. Many diatom species, although not all, have genes from several clades [25, 26]. To date, the whole genomes of the following diatoms have been sequenced: *Thalassiosira pseudonana* [27], *Phaeodactylum tricornutum* [28], *Thalassiosira oceanica* [29], *Pseudo-nitzschia multiseries* [30], *S*. *acus* subsp. *radians* [31], *Fistulifera solaris* [32], *Cyclotella cryptica* [33], *Pseudo-nitzschia multistriata* [34] and *Fragilariopsis cylindrus* [35].

Besides the regular *SIT* genes described above, some genomes were found to contain multi-*SIT* genes which encode several SIT proteins merged head-to-tail within a single reading frame 46 such genes were identified to date. They are present in marine and freshwater diatoms of all three classes: centric, multipolar centric and pennate, as well as a single ciliate species [25, 26, 36].

The phylogenetic analysis of the putative “mature” SIT proteins in multi-SITs, *ie* the sequences produced by cleaving them into fragments with 10 transmembrane domains and all the conservative sites each, in marine diatoms has shown that they all belong to the clade B, a paraphyletic basal clade [25, 26]. According to the authors' hypothesis, this clade has emerged when silica concentration in the ocean was higher than it is now. In modern diatoms, clade B SITs are important under elevated silicic acid concentrations [25].

Two such genes, located in a single chromosome at the distance of 4 kbp from each other, were recently found in a freshwater araphid pennate diatom *Synedra acus* subsp. *radians* from Lake Baikal [36].

In this work we have sequenced three multi-*SIT* genes, as well as 5′- and 3′-ends of their mRNAs, in a freshwater araphid pennate diatom *Synedra ulna* subsp. *danica*. The comparative analysis of the multi-SIT sequences from a range of diatoms has allowed us to detect aspartate-rich conservative motifs (DXDID) which, in our opinion, can serve as the proteolysis sites necessary for processing a multi-SIT into multiple “mature” proteins SIT, each capable of transporting silicic acid. Multi-*SIT*s in the genus *Synedra*, like other multi-*SIT*s, belong to clade B and have arisen from a single non-multiplicate ancestor through a series of duplications.

## Materials and methods

### Diatom culture and DNA isolation

The revision of the genera *Fragilaria*, *Synedra*, *Ulnaria* [37–39] being still in progress, we would rather use in our paper the species names *S*. *ulna* subsp. *danica* (Kütz.) Skabitsch. [40] and *S*. *acus* subsp. *radians* (Kütz.) Skabitsch. [41].

We used a non-axenic culture of *S*. *ulna* subsp. *danica* (Kütz.) Skabitsch. grown from a single cell sampled in Lake Baikal. The cells were cultivated for four weeks at 16°C with intermittent mixing under a natural day-night biorhythm in 20-L glass bottles filled with Diatom Medium (DM) [42].

The cells were then collected on polycarbonate filters (5-μm pores) (Whatman, USA), briefly rinsed with cold DM medium, harvested by centrifugation for 2 min at 16.100 *g* and 4°C and then stored at −70°C.

High-molecular-weight genomic DNA was isolated according to a modification of the method of Jacobs et al. [43] (protocol available at dx.doi.org/10.17504/protocols.io.qh6dt9e).

### Search for *SIT* structural genes in the genome of *S*. *ulna* subsp. *danica*

We developed bidirectional degenerate primers (UniCMLDF–UniCMLDR) (S1 Table) using comparative analysis of the *SIT* structural genes cluster (*SaSIT-TD*, *SaSIT-TRI*) in *S*. *acus* subsp. *radians* (GenBank accession no. KX345281) and the nucleotide sequences encoding the conserved CMLD motifs and their context in *P*.*-n*. *multiseries SIT* (JGI protein ID: 338018), *Achnanthes exigua SIT* (GenBank accession no. EF530636), *Epithemia zebra SIT* (GenBank accession no. EF065521) and *P*. *tricornutum SIT* (GenBank accession no. EU879096) structural genes. PCR products were cloned in the pJET 2.1 vector (Thermo Scientific, USA), and the inserts were sequenced following plasmid DNA isolation with the GeneJET Plasmid DNA Purification Kit (Thermo Scientific, USA). The primer pairs 1100F-739R and 3403F-276R were used to identify a link amongst the PCR fragments deciphered (S1 Table).

The primer pairs 1657F-92R, 9288F-5561R, 1233F-862R and 3531F-172R (S1 Table) were used to determine the location of the *S*. *ulna* subsp. *danica SIT* structural genes relative to each other. The amplicons were extracted from the reaction mixtures by sorption on the AMPure XP magnetic particles (Agencourt, USA), then directly sequenced by Sanger technique with BigDye 3.1 reagent (Applied Biosystems, USA) and finally analysed with 3130XL Analyzer (Applied Biosystems, USA).

The sequences were published under the GenBank accessions MF971079 (*SuSIT1* and *SuSIT2* genes cluster) and MF971078 (*SuSIT3*) (S1 File).

### Isolation of total RNA

An RNeasy Plant Mini Kit (QIAGEN, Germany) was used to isolate total RNA. The quality of the RNA isolated was assessed by means of an Agilent 2100 Bioanalyser with the RNA 6000 Nano LabChip Kit (Agilent Technologies, USA). Products with an RIN index equal to or higher than eight were used for Step-Out RACE-PCR.

### Sequencing of the 5′- and 3′- termini in the mRNA of the *SuSIT* structural genes in *S*. *ulna* subsp. *danica*

A Mint RACE cDNA Amplification Kit (Evrogen, Russia) was used to sequence the 5′- and 3′- termini in the mRNA of *SuSIT* structural genes in *S*. *ulna* subsp. *daniсa*, according to the manufacturer’s instructions.

Several rounds of 5′- and 3′- Step-Out RACE PCR of the *SuSIT* structural genes of *S*. *ulna* subsp. *daniсa* were performed with different pairs of primers (S2 Table). After each round, the PCR products were analysed by electrophoresis in 1% agarose/ethidium bromide gel in TAE buffer, cloned and sequenced.

### Bioinformatic analysis of nucleotide sequences

Vector NTI 11 Academic software (Invitrogen, USA) was applied to analyse the nucleotide sequences of the PCR products and the cDNA. Alignment of the amino acid and nucleotide sequences was performed with the CLUSTALW algorithm using the BioEdit 7.2.5 [44] and Vector NTI 11 software. TmPred [45] software was used to identify transmembrane domains. Pfam [46] software was used to identify boundaries between the putative “mature” SIT proteins in the predicted amino acid sequences. The putative “mature” SIT proteins had approximately the same length of 440 amino acids, where the Markov model of the domain PF03842 can be plotted.

The maximum likelihood trees of the SIT amino acid sequences were built using RAxML 8.0 [47] under the LG substitution model [48] and gamma rate distribution (gamma parameter = 4). This model was selected using Bayesian information criterion via the “find best DNA/protein model” option in the MEGA 6.0.6 software package [49]. 1000 bootstrap trees [50] were built to estimate the node support.

## Results and discussion

### Identification of the *SIT* structural genes in *S*. *ulna* subsp. *danica*

**Fig 1:**
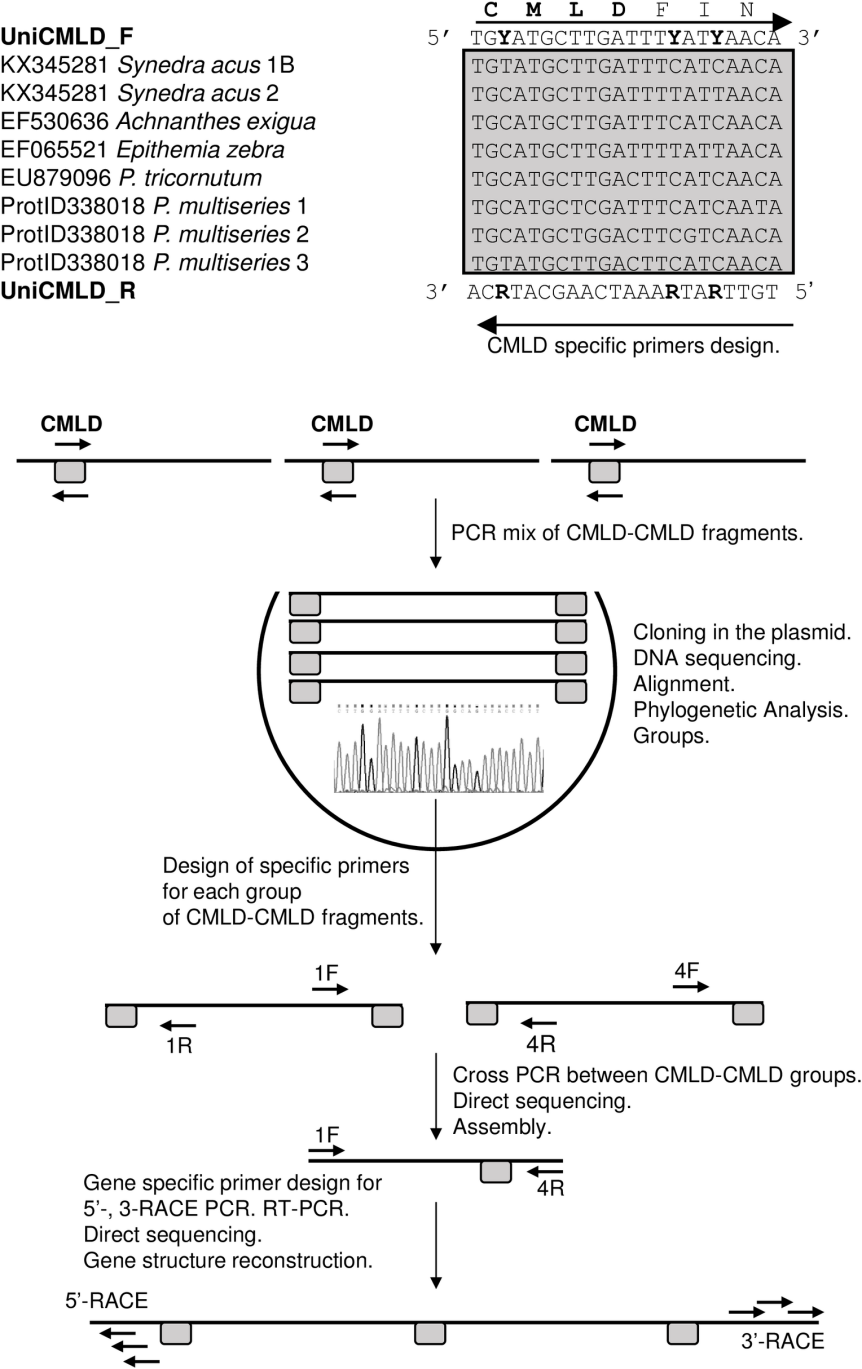
Flow chart of the experiments on deciphering the nucleotide sequences of the *SuSIT* structural genes of *S*. *ulna* subsp. *danica*. Location of the primers is shown by horizontal arrows.

To develop *SIT* structural gene-specific primers, we compared and aligned the nucleotide sequences encoding the CMLD motif of the SIT protein and some amino acids surrounding it (CMLDFIN) for *S*. *acus* subsp. *radians SIT*, *P*.*-n*. *multiseries SIT-TRI*, *A*. *exigua SIT*, *E*. *zebra SIT* and *P*. *tricornutum SIT* (Fig 1). As a direct primer (UniCMLD F), we chose the sequence of the sense DNA chain encoding CMLD and its context, taking into account codon degeneracy and persistence of nucleotide positions. In a similar fashion, we took a complementary antisense sequence of this site as a reverse primer (UniCMLD R). The PCR with these primers and genomic DNA of *S*. *ulna* subsp. *danica* gave us a mix of amplification products, all approximately 1400 nucleotides long, that we separated by cloning. The individual amplification products within the plasmid DNA were isolated, sequenced and compared with the *SIT* structural genes of *S*. *acus* subsp. *radians*. Multiple alignment revealed that the cloned DNA fell into four groups. For each of these groups, we developed unique primers directed towards the CMLD motif. These primers were used to amplify the DNA. This time, the PCR fragments obtained were sequenced directly without cloning. The RACE performed on the total RNA of *S*. *uln*a subs*p*. *danica* and the *SIT* structural gene-specific primers developed enabled us to identify the 5′- and 3′-termini of the correspondin*g SIT* mRNA and to reconstruct the sequences of the *SIT* structural genes. The sequences obtained were verified by direct sequencing of the genomic DNA amplification products (Fig 1). By this approach, we found that *SIT* structural genes are present in the *S*. *ulna* subsp. *danica* genome (we named them *SuSIT1*/*SuSIT2* (GenBank ID MF971079) and *SuSIT3* (GenBank ID MF971078) (S1 File), and each of them encodes a polypeptide chain containing three CMLD motifs.

**Fig 2:**
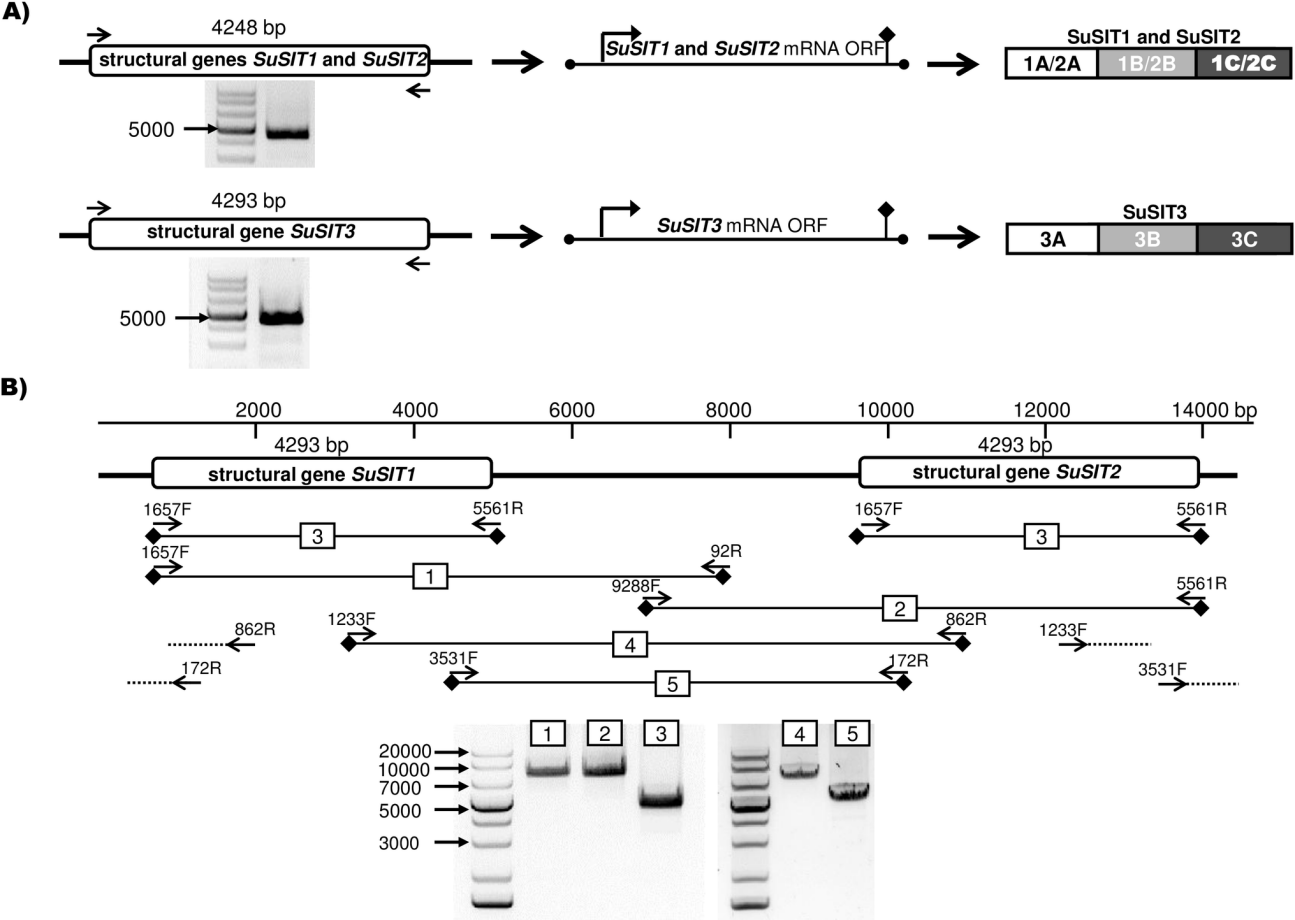
Location of the *SuSIT* structural genes in *S*. *ulna* subsp. *danica*. A) Schemes of the experiment on obtaining *SuSIT1*, *SuSIT2* and *SuSIT3* structural genes. Black solid lines: sequenced DNA and RNA regions, rectangles are the predicted amino acid sequences of SuSIT1, SuSIT2 and SuSIT3 proteins. Numbered A, B and C blocks are the gene regions encoding the assumed “mature” SIT proteins. The electrophoregrams show the uniqueness of the PCR fragments that were later sequences by the Sanger method and localized within the gene. B) Cluster of the *SuSIT1* and *SuSIT2* structural genes: *SuSIT1* and *SuSIT2* are represented with rectangles. Black solid lines show the regions of the *SuSIT* structural genes obtained from the genome DNA. Black arrows are specific primer binding sites. Rectangle with digits represent PCR fragments obtained from corresponding primer pairs.

In order to find a relative position of the *SuSIT* structural genes in *S*. *ulna* subsp. *danica*, we used a combination of terminal primers directed outside the gene. The PCR products from the interior of the gene cannot be amplified under such conditions, whilst the DNA fragments overlapping the site between the *SuSIT* structural genes are being amplified. We found that the genome of *S*. *ulna* subsp. *danica* had three rather than two structural genes encoding long proteins. Each of them contained three CMLD motifs, and two identical genes (*SuSIT1* and *SuSIT2*) connected by a non-coding DNA sequence 5239 bp long (Fig 2). The location of the *SuSIT3* structural gene relative to *SuSIT1* and *SuSIT2* remained unknown.

Schematics of the *S*. *ulna* subsp. *danica SIT* genes are shown in Fig 2. The nucleotide sequence of the *SuSIT1* gene suggests that it can produce a long amino acid sequence, which can be divided into three smaller fragments mapping to the SIT HMM. Each of these fragments contains a single CMLD motif, as well as four GXQ motifs, and is between 436 and 446 amino acid residues in length. It is known that SIT proteins with one conserved CMLD motif (or its analogue) and four GXQ motifs are supposed to transport silicon [14–16]. All these fragments also contain six out of seven conserved amino acids: Q104, N115, H190, Y193, S229 and S372 (positions relative to the PtSIT1 sequence) (S2 File) [51].

Hence, we may propose a hypothesis that each of the three fragments of the *SuSIT1* gene product could be a complete SIT protein able to perform its function, and that SuSIT1 product is posttranslationally cleaved to produce these “mature” proteins. We named these putative “mature” proteins SuSIT1А, SuSIT1В and SuSIT1С, as shown in Fig 2. SuSIT1A and SuSIT1B are highly similar to each other, but relatively distant from SuSIT1C (S3 Table). It is possible that this difference in structure reflects differences in their functions. The sequence, and therefore the structure, of *SuSIT2* is identical to that of *SuSIT1*.

The *SuSIT3* structural gene is also transcribed in a single reading frame and can produce a predicted protein 1416 amino acids long. Three putative “mature” proteins (SuSIT3А, SuSIT3В, and SuSIT3C) 436–446 amino acids long are present in the predicted amino acid sequence.

Having deciphered the *SuSIT* structural genes and identified nine predicted “mature” proteins, we compared them to each other. The conserved CMLD motifs appear to be present in all the “mature” proteins from both SuSIT1/2 and SuSIT3. The same was also true for the conserved GXQ motifs.

**Fig 3:**
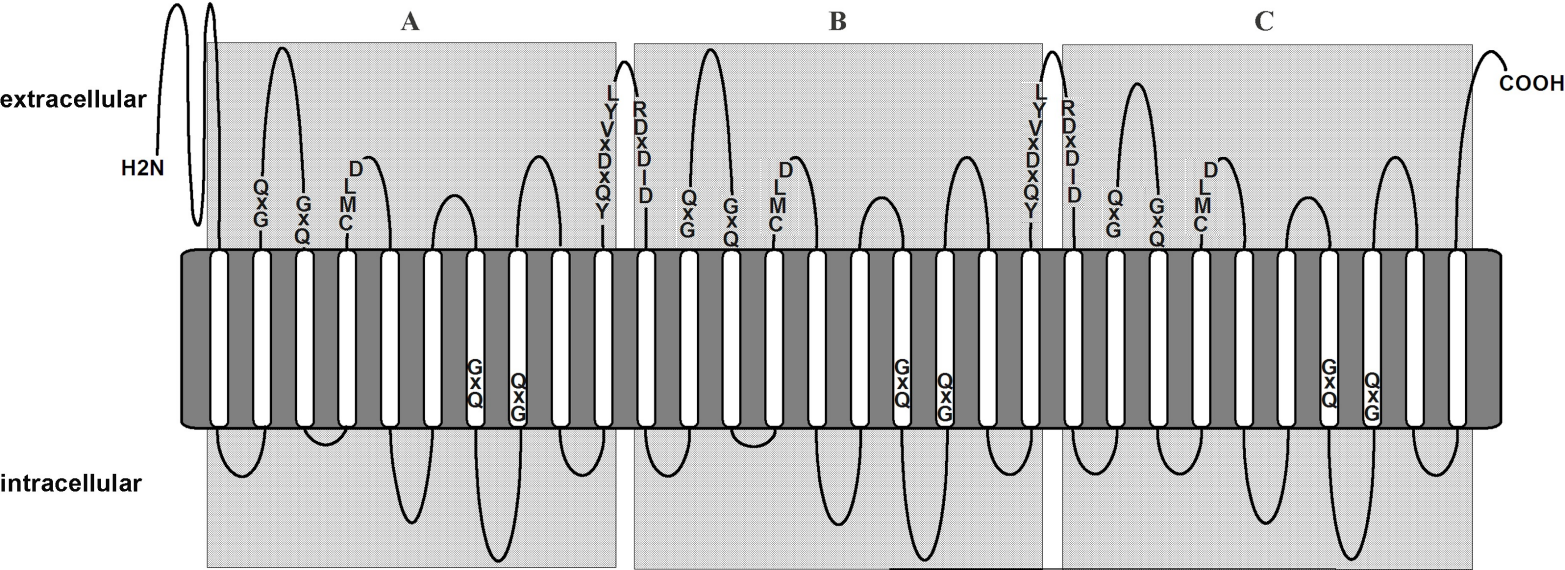
A generalized structure for SuSIT protein in the cytoplasmic membrane. Transmembrane domains (shown as white vertical lines) are based on TMPred predictions. Gray rectangles A, B and C correspond to the SIT domains predicted with Pfam. Conservative motifs GXQ, CMLD, YQXDXVYL and DXDID are shown in the sequence. Labels mark the extracellular and intracellular space, respectively.

The TmPred software showed that each of these “mature” proteins is an integral membrane protein and contains 10 transmembrane domains. The CMLD motif is located at the edge of a hydrophobic region (Fig 3). These data support an argument in favour of the hypothesis that the predicted 436–446-amino-acid-long fragments of *S*. *ulna* subsp. *danica* SITs are “mature” proteins able to fulfil their main function of silicon transport. Identity and similarity data for the predicted SaSIT and SuSIT proteins are given in S3 Table.

Forty-eight full-length predicted amino acid sequences of multi-SIT proteins from 17 diatom species have been published to date [25, 26, 36]. Comparing their sequences, we found conserved motifs with the general formulae YQXDXVYL and DXDID (Fig 3 and S3 File). The latter motif is always located at the boundary of the putative “mature” SIT proteins and absent from the triplicate SIT termini. Thus, it could be a target for the aspartic-acid-specific protease responsible for the posttranslational cleavage of these multi-SITs into “mature” proteins.

Earlier western blotting experiments on the *S*. *acus* subsp. *radians* total protein have detected a polypeptide with a molecular weight of approximately 64 kDa that binds antibodies to the synthetic peptides derived from the SIT amino acid sequence [52]. There is no evidence to the existence of proteins with the molecular weights of 94 or 152 kDa interacting with these antibodies. Antigens of the antibodies to another SIT-derived peptide in several other diatoms’ proteomes (*Thalassiosira pseudonana*, *Skeletonema costatum*, *Chaetoceros gracilis*, *Cyclotella meneghianana*, *Cylindrotheca fusiformis*, *Ditylum brightwellii*, *Nitzschia pelliculoza*, *Phaeodactylum tricornutum*) had weights of 55–64 kDa [53]. DXDID motifs of multi-SITs aren’t flanked by proline residues which could potentially bend the protein secondary structure and thus prevent caspase binding. This fact can serve as an additional line of evidence for their role as the proteolysis site.

**Fig 4:**
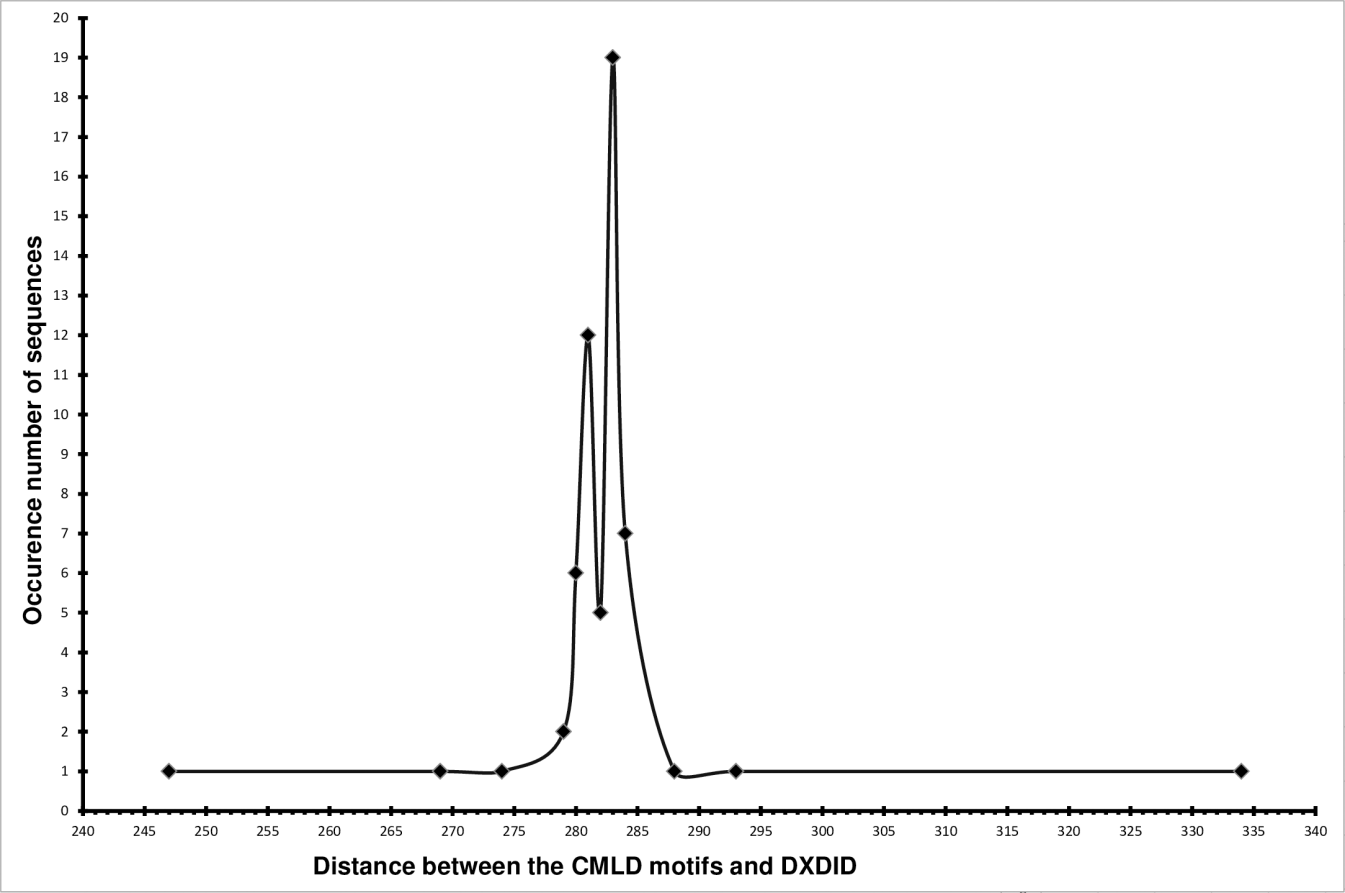
The distribution of distances between the cysteine of CMLD motif and the first amino acid of DXDID motif (or their analogues) in diatom multi-SITs.

Support for this hypothesis also comes from the fact that the distances between the CMLD and DXDID motifs vary in a very narrow range for most diatoms (Fig 4). It suggests that their relative location in the three-dimensional structure of the protein is conserved as well, and thus that these motifs have a conserved function in all the multi-SITs.

On the other hand, we cannot exclude the possibility that the triplicate proteins are functional without being processed. Another conserved motif, YQXDXVYL, is present in all “mature” *S*. *ulna* subsp. *danica* SIT proteins. For the moment, we have no idea what the function of this motif could be.

The aspartic acid-rich sites are well known in the literature as targets for the caspase family of proteases [54]. Although they are mostly known for their role in cell death processes, proteins of this family were shown to be expressed constitutively in the genome of *Thalassiosira pseudonana* [55]. It is possible that they take part in the processing of multi-SIT or other similar multiplicated proteins.

Durkin et al. examined phylogeny of approximately 400 SITs deciphered before 2016 from more than 100 diatom species and from a small number of other silicifying organisms [26]. Many predicted SIT proteins appeared to consist of 2–3 merged monomeric proteins. Each of these monomers contains one conserved CMLD motif and four conserved GXQ motifs, as do “mature” proteins within the SaSIT and SuSIT proteins (S2 File). In the S2 File, we are presenting amino acid sequences of the SITs that were split *in silico* using Pfam software and aligned in order to measure their identity and similarity. There are data concerning *S*. *ulna* subsp. *danica* SITs from the present study, as well as data on *S*. *acus* subsp. *radians* SITs that were published previously [36] The sequences of 87 predicted “mature” SIT proteins are present in S2 File. The CMLD motif was absent only from three proteins (two out of seven *Extubocellulus spinifer* proteins, one out of six *Leptocylindrus danicus* proteins). We would like to note a high identity of localization of many separate amino acid residues in these proteins. For example, the G residue occur in the positions 79, 192, 228, 363, 451, 497, the absolutely conserved W residue occur in the position 466, whilst the L residue is present in the positions 67, 119, 194, 459. The phylogenetic distances between the diatoms from the S2 File are high. This could indicate that some homologous amino acid residues did not change during the evolution. These conserved amino acid residues are likely to participate either in correct folding of tertiary structures or directly in silicon transport.

We found that forty-three complete multi-SIT proteins had the same predicted targets of processing protease (DXDID) as SuSIT and SaSIT do at the boundaries between “monomeric” SITs. Their locations relative to each other are somewhat different, and they are absent from the S3 File.

It is possible that the highly conserved sequences of the predicted “mature” SIT proteins are an essential structure, which perform silicon transport in diatoms. Their more detailed study could possibly shed new light on the silicon transport mechanism.

Since the predicted “mature” SIT proteins within the triplicate and duplicate protein-coding genes of the two *Synedra* species have high sequence identity to each other, we assume that they have emerged in a relatively recent series of duplication events. Duplication that has separated A/B and C proteins probably predates that between proteins A and B, as evidenced by their higher divergence.

**Fig 5:**
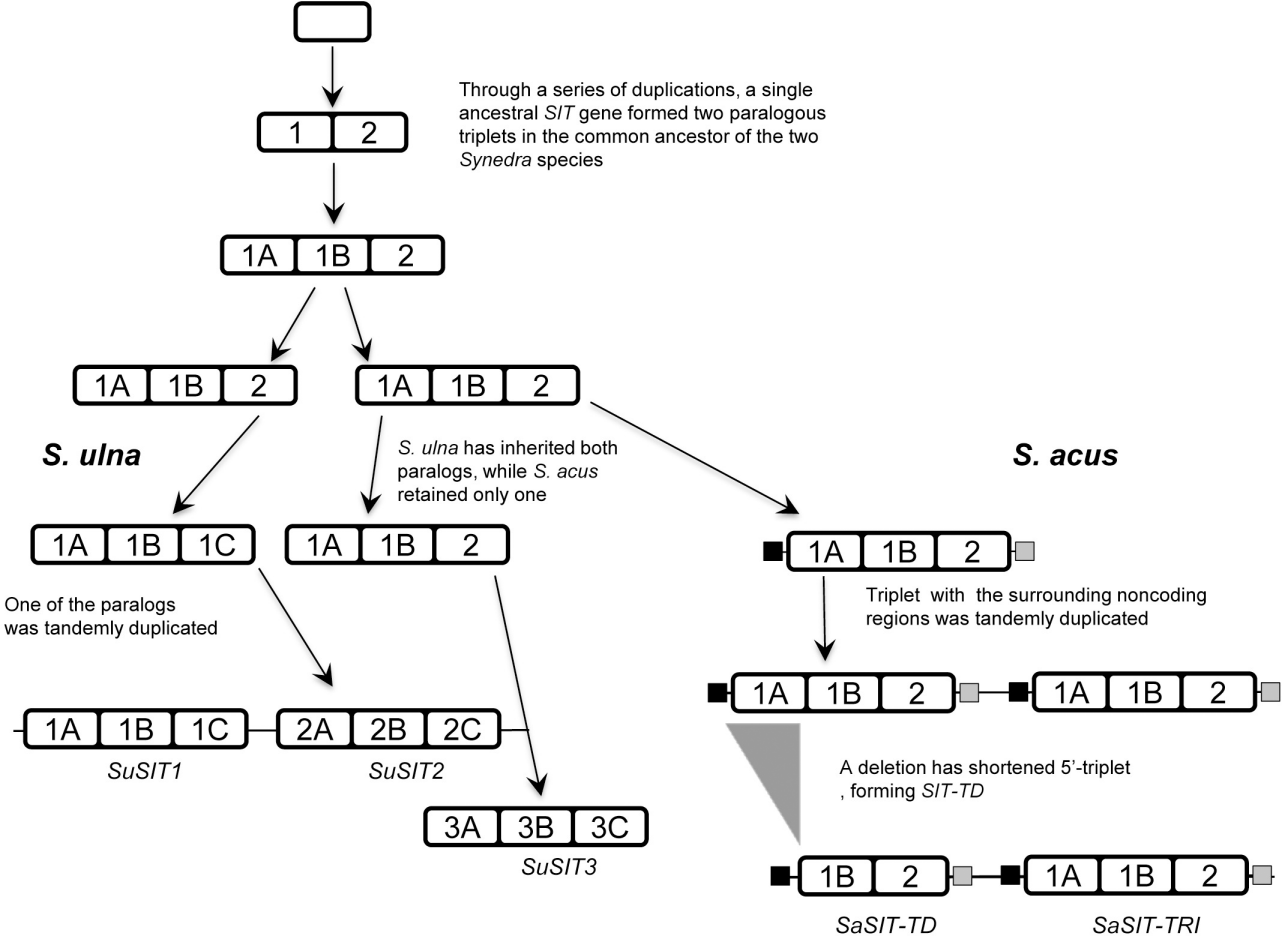
The evolution of the multi-*SIT* genes in the genus *Synedra*. White rectangles are the regions encoding “mature” SIT proteins, black and gray ones are the non-coding flanking repeats.

To elucidate the evolutionary history behind the different SIT proteins of two closely related *Synedra* species, we have performed the phylogenetic analysis. To place our sequences into a broader context, their “mature” proteins were aligned to the SITs from the earlier work [26], and a maximum likelihood tree was built. This tree (S1 Fig) was similar in topology to earlier work [25, 26] and included all five clades. Like the “mature” SIT proteins from the multi*-*SITs of marine diatoms, *Synedra* sequences belonged to clade B. They formed a single clade, within it, which means that they have a single non-duplicate ancestor not shared with other sequenced SITs (Fig 5 and S1 Fig). This ancestral gene was tandemly duplicated in the genome of these two species′ common ancestor, creating a structure not unlike *SaSIT-TD*. Its 5′ region was duplicated further, forming a triplicate ancestor to all modern *Synedra* multi-*SIT*s. This ancestor was duplicated again, though this time it formed two separate paralogs instead of a single multidomain gene. During the species divergence *S*. *ulna* subsp. *danica* has retained both paralogs, one of which has given rise to *SuSIT1* and *SuSIT2*, while the other became *SuSIT3*. *S*. *acus* subsp. *radians*, on the other hand, has lost one of the ancestral paralogs, but duplicated another one (together with the flanking sequences). This duplication explains the neighbouring positions of *SaSIT-TRI* and *SaSIT-TD*. Finally, the 5′-paralog has lost its *SIT1A* and *SIT1B* part in the deletion, thus becoming *SaSIT-TD* (Fig 5).

Of special interest is the absolute identity of the nucleotide sequences of the *SuSIT1* and *SuSIT2* structural genes. Such identity could be a result of a very recent duplication, in particular non-crossover gene conversion [56]. **Gene conversion** is the process by which a gene replaces a homologous gene such that the genes become identical after the conversion event [57]. The mechanism has not yet been fully understood. In a recent review Korunes and Noor [56], point out that “gene conversion has major evolutionary consequences, ranging from immediate effects on nucleotide diversity to long-term consequences that shape genome evolution, species formation, and species persistence” [56].

To our knowledge, non-crossover gene conversion haven't yet been found in diatom genomes.

## Supporting information

S1 Table Amplification conditions used in the study. (DOCX)

S2 Table RACE-PCR conditions for the *S*. *ulna* subsp. *danica* structural *SIT* genes. (DOCX)

S3 Table Identity/similarity comparison of the “mature” SIT proteins of *S*. *uln*a subsp. *danic*a and *S*. *acus* subsp. *radian*s (%). (DOCX)

S1 File Sequences of the *SuSIT1* and S*uSIT2* genes cluster and gene *SuSIT3*. (FASTA)

S2 File Multiple alignment of amino acid sequences of “mature” SITs obtained by *in silico* dissection of duplicated and triplicated predicted proteins of different diatom species. Abbreviations Su ‒ *Synedra ulna* subsp. *danica*; Sa ‒ *Synedra acus* subsp. *radians*; Pd ‒ *Pseudo-nitzschia delicatissima*; Pa ‒ *Pseudo-nitzschia arenysensis*; Ns ‒ *Nitzschia sp*; Np ‒ *Nitzschia punctata*; Pp ‒ *Pseudo-nitzschia pungens*; Pau ‒ *Pseudo-nitzschia australis*; Cw ‒ *Coscinodiscus wailesii*; Ld ‒ *Leptocylindrus danicus*; Ch ‒ *Corethron hystix*; Mp ‒ *Minutocellus polymorphus*; Es ‒ *Extubocellus spinifer*; Ac ‒ *Amphora coffeaeformis*; Cc ‒ *Chaetoceros cf*.; Pm ‒ *Pseudo-nitzschia multiseries*.
(FASTA)

S3 File Multiple alignment of amino acid sequences of multi-SITs. Abbreviations Su ‒ *Synedra ulna* subsp. *danica*; Sa ‒ *Synedra acus* subsp. *radians*; Pd ‒ *Pseudo-nitzschia delicatissima*; Pa ‒ *Pseudo-nitzschia arenysensis*; Ns ‒ *Nitzschia sp*; Np ‒ *Nitzschia punctata*; Pp ‒ *Pseudo-nitzschia pungens*; Pau ‒ *Pseudo-nitzschia australis*; Cw ‒ *Coscinodiscus wailesii*; Ld ‒ *Leptocylindrus danicus*; Ch ‒ *Corethron hystix*; Mp ‒ *Minutocellus polymorphus*; Es ‒ *Extubocellus spinifer*; Ac ‒ *Amphora coffeaeformis*; Cc ‒ *Chaetoceros cf*.; Pm ‒ *Pseudo-nitzschia multiseries*.
(FASTA)

S1 Fig A Maximum likelihood tree of all available SIT proteins at least 400 amino acids long. The multi-SIT proteins were split into “mature” proteins using Pfam. Phylogenetic analysis was performed with RaxML 8.0 using the LG substitution matrix.
(PDF)

## References

[1] Monroe JS, Wicander R. The changing earth: exploring geology and evolution, 6th ed Cengage Learning-Wadsworth, Belmont, CA; 2011.

[2] Iler RK. The Chemistry of Silica: Solubility, Polymerization, Colloid and Surface Properties, and Biochemistry New York, NY: Wiley;1979.

[3] Frings PJ, Clymans W, Fontorbe G, De La Rocha CL, Conley DJ. The continental Si cycle and its impact on the ocean Si isotope budget. Chemical Geology. 2016; 425: 12–36. 10.1016/j.chemgeo.2016.01.020

[4] Pondaven P, Ragueneau O, Tréguer P, Hauvespre A, Dezileau L, Reyss JL. Resolving the ‘opal paradox’ in the Southern Ocean. Nature. 2000; 405: 168–172. 10.1038/35012046 10821269

[5] Sutton JN, André L, Cardinal D, Conley DJ, de Souza GF, Dean J, et al A Review of the Stable Isotope Bio-geochemistry of the Global Silicon Cycle and Its Associated Trace Elements. Frontiers of Earth Science. 2018; 5 112 10.3389/feart.2017.00112

[6] Carlisle EM. Silicon in bone formation. In Silicon and siliceous structures in biological systems Springer, New York, NY 1981 p. 69–94.

[7] Epstein E. Silicon. Annual Review of Plant Physiology and Plant Molecular Biology. 1999; 50: 641–664. Available from: https://www.annualreviews.org/doi/abs/10.1146/annurev.arplant.50.1.641. 15012222 10.1146/annurev.arplant.50.1.641

[8] Knoll AH. Biomineralization and evolutionary history. Reviews in Mineralogy and Geochemistry. 2003; 54: 329–356. Available from: https://www.researchgate.net/profile/Andrew_Knoll/publication/250129981_Biomineralization_and_Evolutionary_History/links/54bbc6b40cf29e0cb04be1a6.pdf.

[9] Round FE, Crawford RM, Mann DG. Diatoms: biology and morphology of the genera Bristol: Cambridge University Press, 1990.

[10] Tréguer PJ, De La Rocha CL. The world ocean silica cycle. Annual Review of Marine Science. 2013; 5: 477–501. 10.1146/annurev-marine-121211-172346 22809182

[11] Tréguer P, Bowler C, Moriceau B, Dutkiewicz S, Gehlen M, Aumont O, et al Influence of diatom diversity on the ocean biological carbon pump. Nature Geoscience. 2018; 11: 1–27. 10.1038/s41561-017-0028-x

[12] Hildebrand M, Volcani BE, Gassman W, Schroeder JI. A gene family of silicon transporters. Nature. 1997; 385: 688–689. 10.1038/385688b0 9034185

[13] Hildebrand M. Biological processing of nanostructured silica in diatoms. Progress in Organic Coatings. 2003; 47: 256–266. 10.1016/S0300-9440(03)00142-5

[14] Grachev MA, Denikina NN, Belikov SI, Likhoshvai EV, Usoltseva MV, Tikhonova IV, et al Elements of the active center of silicon transporters in diatoms. Molecular Biology. 2002; 36: 534–536. Available from: https://link.springer.com/content/pdf/10.1023/A:1019860628910.pdf. 12173473

[15] Sherbakova TA, Masyukova YuA, Safonova TA, Petrova DP, Vereshagin AL, Minaeva TV, et al Conserved motif CMLD in silicic acid transport proteins of diatoms. Molecular Biology. 2005; 39: 269–280. 10.1007/s11008-005-0038-4

[16] Thamatrakoln K, Alverson AJ, Hildebrand M. Comparative sequence analysis of diatom silicon transporters: toward a mechanistic model of silicon transport. Journal of Phycology. 2006; 42: 822–834. 10.1111/j.1529-8817.2006.00233.x

[17] Alverson AJ. Strong purifying selection in the silicon transporters of marine and freshwater diatoms. Limnology and Oceanography. 2007; 52: 1420–1429. 10.4319/lo.2007.52.4.1420

[18] Sapriel G, Quinet M, Heijde M, Jourden L, Tanty V, Luo G, et al Genome-wide transcriptome analyses of silicon metabolism in *Phaeodactylum tricornutum* reveal the multilevel regulation of silicon acid transporters. PLoS ONE. 2009; 10: 189–195. 10.1371/journal.pone.0007458 PMC2758714 19829693

[19] Keeling PJ, Burki F, Wilcox HM, Allam B, Allen EE, Amaral-Zettler LA, et al The Marine Microbial Eukaryote Transcriptome Sequencing Project (MMETSP): illuminating the functional diversity of eukaryotic life in the oceans through transcriptome sequencing. PLoS Biology. 2014; 12(6): e1001889 10.1371/journal.pbio.1001889 24959919 PMC4068987

[20] Kang LK, Feng CC, Chang J, Gong GC. Diversity and expression of diatom silicon genes during a flood eventin the East china Sea. Marine Biology. 2015; 39: 270–281. 10.1007/s00227-015-2687-8

[21] Likhoshway YV, Masyukova YA, Sherbakova TA, Petrova DP, Grachev MA. Detection of the gene responsible for silicic acid transport in Chrysophycean algae. Doklady Biological Sciences. 2006; 408: 256–260. . 16909993 10.1134/s001249660603015x

[22] Marron AO, Alston MJ, Akam M, Caccamo M, Holland PWH, Walker G. A family of diatom-like silicon transporters in the siliceous loricate choanoflagellates. Archive of “Proceedings of the Royal Society B: Biological Sciences”. 2013; 280: 21122543. 10.1098/rspb.2012.2543 23407828 PMC3574361

[23] Durak GM, Taylor AR, Walker CE, Probert I, Vargas C, Audic S, et al A role for diatom-like silicon transporters in calcify coccolithophores. Nature Communications. 2016 10.1038/ncomms10543 26842659 PMC4742977

[24] Thamatrakoln K, Alverson AJ, Hildebrand M. Comparative sequence analysis of diatom silicon transporter: taward a mechanistic model of silicon transport. The Journal of Physiology. 2006; 42: 822–834. 10.1111/j.1529-8817.2006.00233.x

[25] Durkin CA, Marchetti A, Bender SJ, Truong T, Morales R, Mock T, et al Frustule-related gene transcription and the influence of diatom community composition on silica precipitation in an iron-limited environment. Limnology and Oceanography. 2012; 57: 1619–1633. 10.4319/lo.2012.57.6.1619

[26] Durkin CA, Koester JA, Bender SJ, Armbrust EV. The evolution of silicon transporters in diatoms. Journal of Phycology. 2016; 52: 716–731. 10.1111/jpy.12441 27335204 PMC5129515

[27] Armbrust EV, Berges JA, Bowler C, Green BR, Martinez D, Putnam NH, et al The genome of the diatom *Thalassiosira pseudonana*: Ecology, evolution, and metabolism. Science. 2004; 306: 1–33. 10.1126/science.1101156 15459382

[28] Bowler C, Allen AE, Badger JH, Grimwood J, Jabbari K, Kuo A, et al The *Phaeodactylum* genome reveals the evolutionary history of diatom genomes. Nature. 2008; 456: 239–244. 10.1038/nature07410 18923393

[29] Lommer M, Specht M, Roy AS, Kraemer L, Gutowska MA, Wolf J, et al Genome and low-iron response of an oceanic diatom adapted to chronic iron limitation. Genome Biology. 2012 10.1186/gb-2012-13-7-r66 22835381 PMC3491386

[30] Mukherjee S, Stamatis D, Bertsch J, Ovchinnikova G, Verezemska O, Isbandi M, et al Genomes OnLine Database (GOLD) v.6: data updates and feature enhancements. Nucleic Acids Research. 2017; 45: D446–D456. 10.1093/nar/gkw992 27794040 PMC5210664

[31] Galachyants YP, Zakharova YR, Petrova DP, Morozov AA, Sidorov IA, Marchenkov AM, et al Sequencing of the complete genome of an araphid pennate diatom *Synedra acus* subsp. *radians* from Lake Baikal. Doklady Biochemistry and Biophysics. 2015; 461: 84–88. 10.1134/S1607672915020064 25937221

[32] Tanaka T, Maeda Y, Veluchamy A, Tanaka M, Abida H, Maréchal E, et al Oil accumulation by the oleaginous diatom *Fistulifera solaris* as revealed by the genome and transcriptome. Plant Cell. 2015; 27: 162–172. 10.1105/tpc.114.135194 25634988 PMC4330590

[33] Traller JC, Cokus SJ, Lopez DA, Gaidarenko O, Smith SR, McCrow JP, et al Genome and methylome of the oleaginous diatom *Cyclotella cryptica* reveal genetic flexibility toward a high lipid phenotype. Biotechnology for Biofuels. 2016; 20169(258). 10.1186/s13068-016-0670-3 PMC5124317 27933100

[34] Basu S, Patil S, Mapleson D, Russo MT, Vitale L, Fevola C, et al Finding a partner in the ocean: molecular and evolutionary bases of the response to sexual cues in a planktonic diatom. New Phytologist. 2017; 215: 140–156. 10.1111/nph.14557 28429538 PMC5485032

[35] Mock T, Otillar RP, Strauss J, McMullan M, Paajanen P, Schmutz J, et al Evolutionary genomics of the cold-adapted diatom *Fragilariopsis cylindrus*. Nature. 2017; 541: 536–540. 10.1038/nature20803 28092920

[36] Marchenkov AM, Bondar AA, Petrova DP, Habudaev KV, Galachyants YuP, Zakharova YuR, et al Unique configuration of genes of silicon transporter in the freshwater pennate diatom *Synedra acus* subsp. *radians*. Doklady Biochemistry and Biophysics. 2016; 471: 407–409. 10.1134/S1607672916060089 28058681

[37] Williams DM, Round FE. Revision of the genus *Fragilaria*. Diatom Research. 1988; 2: 267–288. 10.1080/0269249X.1987.9705004

[38] Bukhtiyarova LN, Compère P. New taxonomical combinations in some genera of Bacillariophyta. Algologia. 2006; 2: 280–283.

[39] Kulikovskiy M, Lange–Bertalot H, Annenkova N, Gusev E, Kociolek JP. Morphological and molecular evidence support description of two new diatom species from the genus *Ulnaria* in Lake Baikal. Fottea, Olomouc. 2016; 16: 34–42. 10.5507/fot.2015.011

[40] Skabichevskii AP. Notulae systematicae e sectione cryptogamica institute botanici nomine VL Komarovi academiae scientiarum URSS. 1959; 12: 46–57. In Russian].

[41] Skabichevskii AP. Planctonic diatoms of the freshwaters of the USSR: systematics, ecology and distribution Moscow: Izd-vo MGU, 1960. [In Russian].

[42] Thompson AS, Rhodes JC, Pettman I. Culture collection of algae and protozoa, catalogue of strains. 5th ed. Cumbria;1988.

[43] Jacobs JD, Ludwig JR, Hildebrand M, Kukel A, Feng TY, Ord RW, et al Characterization of two circular plasmids from the marine diatom *Cylindrotheca fusiformis*: plasmids hybridize to chloroplast and nuclear DNA. Molecular and General Genetics. 1992; 233: 302–310. 10.1007/BF00587592 1603070

[44] Hall TA. BioEdit: A user-friendly biological sequence alignment editor and analysis program for Windows 95/98/NT. Nucleic Acids Symposium Series. 1999; 41: 95–98.

[45] Hofmann K, Stoffel W. TMbase–A database of membrane spanning proteins segments. Biological Chemistry. Hoppe-Seyler. 1993; 374: 166.

[46] Finn RD, Coggill P, Eberhardt RY, Eddy SR, Mistry J, Mitchell AL, Bateman A. The Pfam protein families database: towards a more sustainable future. Nucleic Acids Research. 2016; 44: D279–D285. 10.1093/nar/gkv1344 26673716 PMC4702930

[47] Stamatakis A. RAxML version 8: a tool for phylogenetic analysis and post-analysis of large phylogenies. Bioinformatics. 2014; 30: 1312–1313. 10.1093/bioinformatics/btu033 24451623 PMC3998144

[48] Le SQ, Gascuel O. An Improved General Amino Acid Replacement Matrix. Molecular Biology and Evolution. 2008; 25: 1307–1320. 10.1093/molbev/msn067 18367465

[49] Tamura K, Stecher G, Peterson D, Filipski A, Kumar S. MEGA6: Molecular Evolutionary Genetics Analysis version 6.0. Molecular Biology and Evolution. 2013; 30: 2725–2729. 10.1093/molbev/mst197 24132122 PMC3840312

[50] Zuckerkandl E, Pauling L. Evolutionary divergence and convergence in proteins, in evolving genes and proteins. In: Bryson V, Vogel HJ, editors. Evolving Genes and Proteins Academic Press, New York; 1965 p. 97–166. 10.1016/B978-1-4832-2734-4.50017–6

[51] Knight MJ, Senior L, Nancolas B, Ratcliffe S, Curnow P. Direct evidence of the molecular basis for biological silicon transport. Nature Communications. 2016; 7: 11926 10.1038/ncomms11926 27305972 PMC4912633

[52] Petrova DP, Bedoshvili YD, Shelukhina IV, Samukov VV, Korneva ES, Vereshagin AL, et al Detection of the silicic acid transport protein in the freshwater diatom *Synedra acus* by immunoblotting and immunoelectron microscopy. 2007; 417(1): 295–298. 10.1134/S1607672907060014 18274442

[53] Thamatrakoln K. Hildebrand M. Analysis of *Thalassiosira pseudonana* silicon transporters indicates distinct regulatory levels and transport activity through the cell cycle. Eukaryotic Cell. 2007; 6(2): 271–279. 10.1128/EC.00235-06 17172435 PMC1797941

[54] Cryns V, Yuan J. Proteases to die for. Genes and Development. 1998; 11:1551–70. 10.1101/gad.12.11.1551 9620844

[55] Bidle KD, Bender SJ. Iron starvation and culture age activate metacaspases and programmed cell death in the marine diatom *Thalassiosira pseudonana*. Eukaryotic Cell. 2008; 2: 223–236. 10.1128/EC.00296-07 PMC2238155 18039944

[56] Korunes KL, Noor MA. Gene conversion and linkage: effects on genome evolution and speciation. Molecular Ecology. 2017; 26(1): 351–364. 10.1111/mec.13736 27337640

[57] Hastings PJ. Mechanisms of ectopic gene conversion. Genes. 2010; 1(3): 427–439. 10.3390/genes1030427 24000309 PMC3758752

