# Supplementary material for: A family of silicon transporter structural genes in a pennate diatom Synedra ulna subsp. danica (Kütz.) Skabitsch
Source: PLoS One. 2018 Aug 29;13(8):e0203161. doi: 10.1371/journal.pone.0203161 (PMC6114903; doi:10.1371/journal.pone.0203161)
Supplement: S1 Table — (DOCX) [file pone.0203161.s001.docx]

S1 Table. Amplification conditions used in the study.

| **Pair primers** | **Sequences (5'-3')** | **Polymerase** | **PCR conditions** |
| --- | --- | --- | --- |
| UniCMLD F  UniCMLD R | TGYATGCTTGATTTYATYAACA | Phusion Hot Start II (Thermo Scientific, USA) | 98 °С − 2 min, 40 cycles (98 °С‒15 sec, 57 °С‒2 min, 72 °С‒5 min), 72 °С ‒7 min. |
|  | TGTTRATRAAATCAAGCATRCA |  |  |
| 1100F  739R | CGCGTGCGTTGTTGAATACG | Q5 Hot Start High-Fidelity (NEB, USA) | 98 °С − 1 min, 35 cycles (98 °С‒10 sec, 57 °С‒15 sec, 72 °С − 10 min), 72 °С ‒10 min. |
|  | CTCCAGAGCAAATTCCGATAGAT |  |  |
| 1657F  92R | CGAAAATTAGAAAATTCACTCTCTTCC | Q5 Hot Start High-Fidelity (NEB, USA) | 98 °С − 1 min, 35 cycles (98 °С‒10 sec, 62 °С‒15 sec, 72 °С‒10 min), 72 °С ‒10 min. |
|  | TCCTTCAATGCCACGTTAGCT |  |  |
| 9288F  5561R | CAAGCTAGTCTAGCATCTCATTCTGTC | Q5 Hot Start High-Fidelity (NEB, USA) | 98 °С − 1 min, 35 cycles (98 °С − 10 sec, 62 °С − 15 sec, 72 °С − 10 min), 72 °С − 10 min. |
|  | GATAAAGATCTGTGTATGACAAACTAACTAC |  |  |
| 1233F  862R | AGGTCTGAATGACACAGTAACCGAA | Q5 Hot Start High-Fidelity (NEB, USA) | 98 °С − 1 min, 35 cycles (98 °С‒10 sec, 63 °С‒15 sec, 72 °С‒10 min), 72 °С ‒10 min. |
|  | GGCGGTCAATATCAGCGTCA |  |  |
| 3531F  172R | TGAATTGGAAGTCGAGGCATAA | Q5 Hot Start High-Fidelity (NEB, USA) | 98 °С − 1 min, 35 cycles (98 °С − 10 sec, 60 °С − 15 sec, 72 °С − 10 min), 72 °С − 10 min. |
|  | CAGCACCTTCGTTTCAGATGTC |  |  |
