# Supplementary material for: A family of silicon transporter structural genes in a pennate diatom Synedra ulna subsp. danica (Kütz.) Skabitsch
Source: PLoS One. 2018 Aug 29;13(8):e0203161. doi: 10.1371/journal.pone.0203161 (PMC6114903; doi:10.1371/journal.pone.0203161)
Supplement: S2 Table — (DOCX) [file pone.0203161.s002.docx]

**S2 Table. RACE-PCR conditions for structural *SIT* genes *S. ulna* subsp. *danica*.**

| Application | GSP (Gene Specific Primers) for RACE PCR and their structure, I-III rounds. | t- annealing primers in, °C | Number of cycles in the reaction |
| --- | --- | --- | --- |
| 5′‒RACE PCR *SuSIT1* and *SuSIT2* *S. ulna* subsp. *danica* | I) 172R (CAGCACCTTCGTTTCAGATGTC) | 60 | 30 |
|  | II) 97R (GCCTGTTTCATCAGGGACATTCA) | 57 | 18 |
|  | III) 50R (TGTGAGCATAAGGATTGCTAGATG) | 57 | 18 |
| 3′‒RACE PCR *SuSIT1* and *SuSIT2* *S. ulna* subsp. *danica* | I) 941F (CGAAGCAGACTAAGATCTCTAAAGAT) | 64 | 28 |
|  | II) 1083F (GGACTCACATAAGATAACGCATGTC) | 64 | 18 |
|  | III) 1233F (AGGTCTGAATGACACAGTAACCGAA) | 62 | 18 |
| 5′‒RACE PCR *SuSIT3* *S. ulna* subsp. *danica* | I) 241 R (GCAGGGTGGAGGCCGAAA) | 60 | 26 |
|  | II) 163R (TGGACAATAATCAAAGAGAGGATGA) | 60 | 18 |
|  | III) 153R (TCAAAGAGAGGATGAGCAATACAG) | 60 | 18 |
| 3′‒RACE PCR *SuSIT3* *S. ulna* subsp. *danica* | I) 2744F (CACTTCCGCCCCTGTGAC) | 62 | 26 |
|  | II) 2920F (TTTGGTTCCTTATTGTATGGCT) | 55 | 18 |
|  | III) 3403F (CAAACGAGCCACCTCGCACT) | 60 | 18 |
