# Supplementary material for: A family of silicon transporter structural genes in a pennate diatom Synedra ulna subsp. danica (Kütz.) Skabitsch
Source: PLoS One. 2018 Aug 29;13(8):e0203161. doi: 10.1371/journal.pone.0203161 (PMC6114903; doi:10.1371/journal.pone.0203161)
Supplement: S3 Table — (DOCX) [file pone.0203161.s003.docx]

**S3 Table. Identity/similarity comparison of the mature SIT proteins of *S. ulna* subsp. *danica* and *S. acus* subsp. *radians* (%).**

| **Столбец1** | SuSIT 1A | SuSIT 1B | SuSIT 1C | SuSIT 2A | SuSIT 2B | SuSIT 2C | SuSIT 3A | SuSIT 3B | SuSIT 3C | SaSIT-TRI 1A | SaSIT-TRI 1B | SaSIT-TRI 2 | SaSIT-TD 1B | SaSIT-TD 2 |
| --- | --- | --- | --- | --- | --- | --- | --- | --- | --- | --- | --- | --- | --- | --- |
| SuSIT 1A | ‒ | 92.2/96.3 | 70.5/82.3 | ‒ | 92.2/96.3 | 70.5/82.3 | 95/97.9 | 92.9/96.6 | 71.3/83 | 95.2/97.9 | 92.9/96.6 | 70.6/83.3 | 92.9/96.6 | 70.6/83.3 |
| SuSIT 1B | 92.2/96.3 | ‒ | 69.1/81 | 92.2/96.3 | ‒ | 69.1/81 | 93.2/96.3 | 94.6/69.7 | 70.6/82.6 | 92.4/96.3 | 94.2/97.5 | 69.5/81.9 | 94.2/97.5 | 69.5/81.9 |
| SuSIT 1C | 70.5/82.3 | 69.1/81 | ‒ | 70.5/82.3 | 69.1/81 | ‒ | 70.5/82.3 | 69.1/81 | 87.5/95.2 | 70.6/82.8 | 69.4/81.9 | 88.4/95.7 | 69.4/81.9 | 88.4/95.7 |
| SuSIT 2A | ‒ | 92.2/96.3 | 70/82.6 | ‒ | 92.2/96.3 | 70/82.6 | 95/97.9 | 92.9/96.6 | 71.3/83 | 95.2/97.9 | 92.9/96.6 | 70.6/83.3 | 92.9/96.6 | 70.6/83.3 |
| SuSIT 2B | 92.2/96.3 | ‒ | 68.8/81 | 92.2/96.3 | ‒ | 68.8/81 | 93.2/96.3 | 94.6/69.7 | 70.6/82.6 | 92.4/96.3 | 94.2/97.5 | 69.5/81.9 | 94.2/97.5 | 69.5/81.9 |
| SuSIT 2C | 70.5/82.3 | 69.1/81 | ‒ | 70.5/82.3 | 69.1/81 | ‒ | 70.5/82.3 | 69.1/81 | 87.5/95.2 | 70.6/82.8 | 69.4/81.9 | 88.4/95.7 | 69.4/81.9 | 88.4/95.7 |
| SuSIT 3A | 95/97.9 | 93.2/96.3 | 70.5/82.3 | 95/97.9 | 93.2/96.3 | 70.5/82.3 | ‒ | 97.5/98.6 | 71/83.3 | 98.9/99.5 | 96.8/97.9 | 71.3/84.2 | 96.8/97.9 | 71.3/84.2 |
| SuSIT 3B | 92.9/96.6 | 94.6/69.7 | 69.1/81 | 92.9/96.6 | 94.6/69.7 | 69.1/81 | 97.5/98.6 | ‒ | 70.8/82.8 | 96.3/98.2 | 98/98.9 | 70/82.4 | 98/98.9 | 70/82.4 |
| SuSIT 3C | 71.3/83 | 70.6/82.6 | 87.5/95.2 | 71.3/83 | 70.6/82.6 | 87.5/95.2 | 71/83.3 | 70.8/82.8 | ‒ | 71.3/83.7 | 70.4/83.7 | 97/98.9 | 70.4/83.7 | 97/98.9 |
| SaSIT-TRI 1A | 95.2/97.9 | 92.4/96.3 | 70.6/82.8 | 95.2/97.9 | 92.4/96.3 | 70.6/82.8 | 98.9/99.5 | 96.3/98.2 | 71.3/83.7 | ‒ | 97/98.4 | 70.4/83.3 | 97/98.4 | 70.4/83.3 |
| SaSIT-TRI 1B | 96.8/97.9 | 94.2/97.5 | 69.4/81.9 | 96.8/97.9 | 94.2/97.5 | 69.4/81.9 | 96.8/97.9 | 98/98.9 | 70.4/83.7 | 97/98.4 | ‒ | 69.5/83.1 | ‒ | 69.5/83.1 |
| SaSIT-TRI 2 | 70.6/83.3 | 69.5/81.9 | 88.4/95.7 | 70.6/83.3 | 69.5/81.9 | 88.4/95.7 | 71.3/84.2 | 70/82.4 | 97/98.9 | 70.4/83.3 | 69.5/83.1 | ‒ | 69.5/83.1 | ‒ |
| SaSIT-TD 1B | 92.9/96.6 | 94.2/97.5 | 69.4/81.9 | 92.9/96.6 | 94.2/97.5 | 69.4/81.9 | 96.8/97.9 | 98/98.9 | 70.4/83.7 | 97/98.4 | ‒ | 69.5/83.1 | ‒ | 69.5/83.1 |
| SaSIT-TD 2 | 70.6/83.3 | 69.5/81.9 | 88.4/95.7 | 70.6/83.3 | 69.5/81.9 | 88.4/95.7 | 71.3/84.2 | 70/82.4 | 97/98.9 | 70.4/83.3 | 69.5/83.1 | ‒ | 69.5/83.1 | ‒ |
