# Supplementary figures and images for: A family of silicon transporter structural genes in a pennate diatom Synedra ulna subsp. danica (Kütz.) Skabitsch
Source: PLoS One. 2018 Aug 29;13(8):e0203161. doi: 10.1371/journal.pone.0203161 (PMC6114903; doi:10.1371/journal.pone.0203161)

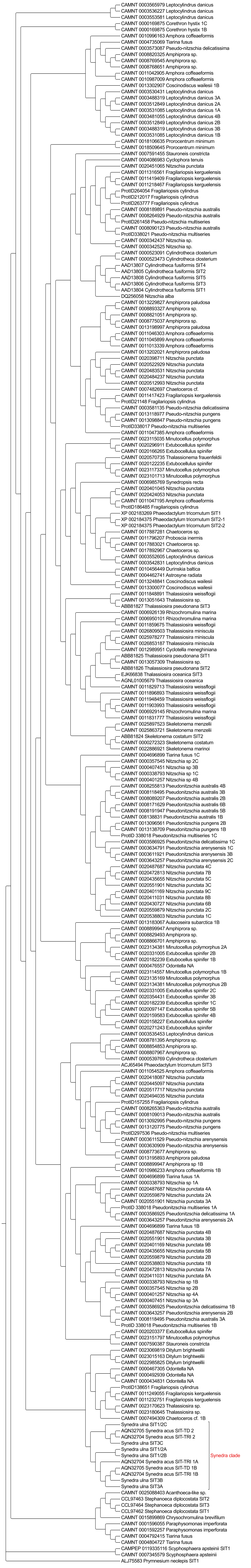

Supplement: S1 Fig — The multi-SIT proteins were split into “mature” proteins using Pfam. Phylogenetic analysis was performed with RaxML 8.0 using the LG substitution matrix. (PDF) [file pone.0203161.s007.pdf]
